# Generating a Cell Model to Study ER Stress in iPSC-Derived Medium Spiny Neurons from a Patient with Huntington’s Disease

**DOI:** 10.3390/ijms26188930

**Published:** 2025-09-13

**Authors:** Vladlena S. Makeeva, Anton Yu. Sivkov, Suren M. Zakian, Anastasia A. Malakhova

**Affiliations:** Institute of Cytology and Genetics of Siberian Branch of the Russian Academy of Sciences, Novosibirsk 630090, Russia; vladamakkeeva@gmail.com (V.S.M.); zakian@bionet.nsc.ru (S.M.Z.)

**Keywords:** iPSC-based cell model, Huntington’s disease, ER stress, XBP1-TagRFP biosensor, iPSC-derived medium spiny neurons

## Abstract

iPSCs and their derivatives are used to investigate the molecular genetic mechanisms of human diseases, to identify therapeutic targets, and to screen for small molecules. Combining technologies for generating patient-specific iPSC lines and genome editing allows us to create cell models with unique characteristics. We obtained and characterized three iPSC lines by reprogramming peripheral blood mononuclear cells of a patient with Huntington’s disease (HD) using episomal vectors encoding Yamanaka factors. iPSC lines expressed pluripotency marker genes, had normal karyotypes and were capable of differentiating into all three germ layers. The obtained iPSC lines are useful for modeling disease progression in vitro and studying pathological mechanisms of HD, such as ER stress. A transgene of genetically encoded biosensor XBP1-TagRFP was introduced into the iPSCs to visualize ER stress state of cells. The study demonstrated that iPSC-derived medium spiny neurons develop ER stress, though the IRE1-mediated pathway does not seem to be involved in the process.

## 1. Introduction

Huntington’s disease (HD) is an autosomal dominant neurodegenerative disorder caused by the expansion of CAG repeats in the first exon of the *HTT* gene. Elongation of the polyglutamine tract of the protein leads to aberrant interactions of mutant huntingtin, the formation of aggregates and N-terminal fragments of the protein, which are toxic to cells [1]. Accumulated information on the disease mechanisms has promoted the development of effective therapeutic agents [2].

To date, there are no approved therapies that will not only alleviate the external symptoms of the disease, such as chorea, cognitive impairment, and emotional disturbance, but also prevent the destruction of neurons [3,4]. A model system that allows the investigation of harmful processes occurring in neurons caused by mutant huntingtin will facilitate the evaluation of potential therapeutic interventions. Animal models differ from human organisms in terms of molecular mechanisms of regulation, signaling pathways, and protein structures. They are not suitable as reliable objects for scientific research [5]. The use of human iPSC cultures not only solves the problem of genomic differences, but also allows the creation of patient-specific models, which is relevant due to the ability of iPSCs to differentiate into specific cell types [6]. Human iPSCs are in a primed pluripotent state. That is, they can give rise to all embryonic structures, except for extraembryonic tissues and organs. They can be used to create organoids and to generate synthetic embryos under certain conditions [7,8,9]. Although HD is triggered by the presence of one mutation in one allele of the *HTT* gene, its phenotypic manifestation depends on both the length of CAG repeats and the genetic background of a particular patient [10,11,12,13]. Among known hallmarks of HD, endoplasmic reticulum (ER) stress is addressed. One of the proposed mechanisms for HD is the binding of mutant HTT and its toxic oligomers with components of the endoplasmic reticulum-associated protein degradation (ERAD) system [14,15,16]. The main participants of the unfolded protein response (UPR) system are the proteins PERK, IRE1, and ATF6 which are anchored to the ER membrane. Protein kinase IRE1 is bound in its inactive form to the chaperone-binding protein BiP. Upon accumulation of unfolded proteins, IRE1 dissociates from the BiP/GRP78 complex and is activated by autophosphorylation. Active IRE1 splices the mRNA encoding the transcription factor XBP1, which is then processed into the active form XBP-1s. XBP-1s translocates into the nucleus, inducing transcription of BiP and other chaperones and target genes associated with ERAD regulation [17]. Indeed, XBP1-deficient mice were more resistant to developing disease features [18].

One of the most effective approaches to detecting ER stress in cell cultures is the use of special biosensors [19]. The XBP1-TagRFP sensor for ER stress [20] associated with the IRE1 pathway was used in a recent study to detect the development of ER stress in transgenic iPSC lines derived from Parkinson’s disease patients [21].

**Figure 1:**
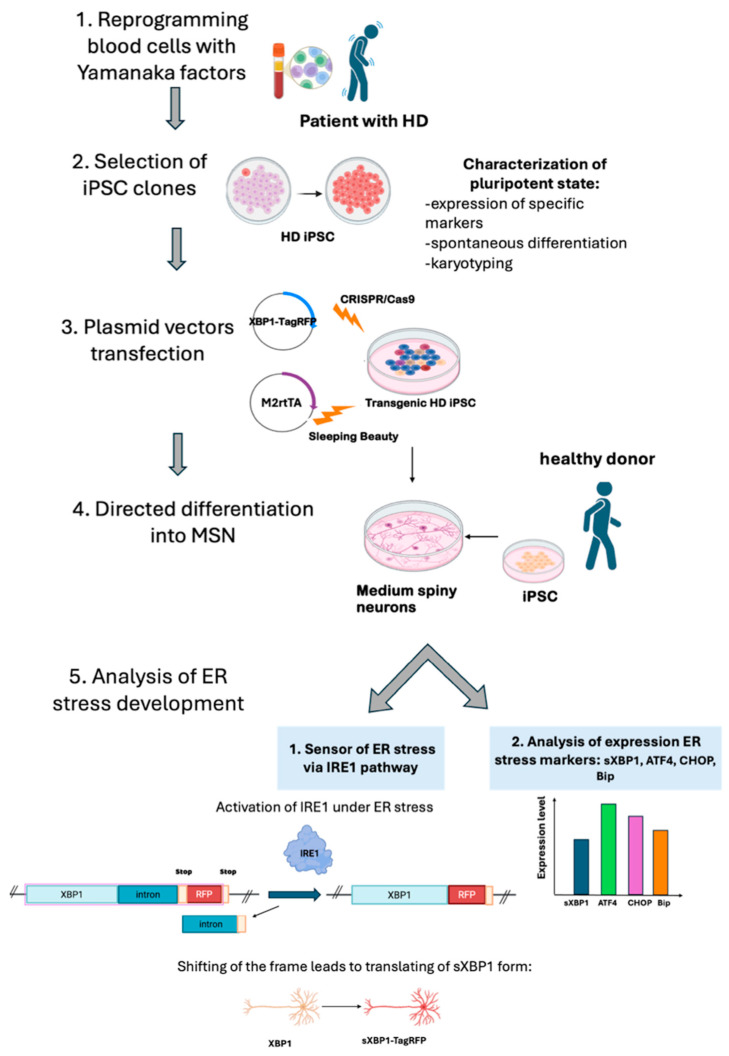
Steps of the experiment: 1. Obtaining peripheral mononuclear blood cells from a patient with Huntington’s disease and reprogramming them into iPSCs by overexpressing Yamanaka factors. 2. Characterizing and selecting iPSC clones. 3. Inserting a transgene coding for the biosensor XBP1-TagRFP using CRISPR/Cas9 and a transgene encoding the transactivator M2rtTA using the Sleeping Beauty method. 4. Directly differentiating transgenic iPSC lines from an HD patient into medium spiny neuron cultures. 5. Analyzing the activity of the XBP1-TagRFP sensor in patient-specific iPSC-derived neuronal cultures grown in an antioxidant-free medium and estimating the expression of specific markers of ER stress in iPSC-derived neurons.

In our work, we obtained iPSC lines from an HD patient with an expanded CAG repeat tract in the *HTT* gene and used them to analyze the development of ER stress along the IRE1 pathway in differentiated neuronal derivatives. For this purpose, the XBP1-TagRFP biosensor was integrated into the iPSCs, which were then differentiated into medium spiny neurons (MSNs) (Figure 1). The biosensor is activated after *XBP1* splicing by the IRE1 protein during ER stress, resulting in a reading frame shift in the mRNA and the expression of the part of the construct encoding for the fluorescent TagRFP protein [20,22]. Thus, we can observe the occurrence of ER stress through red fluorescence [21].

The reproducibility of existing protocols of iPSC-based MSN generation [23,24,25,26,27] has been previously discussed, and some studies have attempted to verify and compare the selected protocols [28]. The researchers of this study analyzed differentiated MSN cultures using electrophysiology, immunostaining and quantitative PCR and highlighted challenges in MSN differentiation, such as low number of MSNs compared to other CNS subtypes (1), and low reproducibility between iPSC differentiation protocols (2).

We aimed to assess the capability of the obtained iPSC lines to differentiate into MSNs. We used several differentiation protocols, as each one has its own limitations [25,26,27]. There are various approaches to neuronal differentiation [29]. Two methods have been highlighted in the literature—direct differentiation via the induction of expression of specific factors, and indirect differentiation mediated by signaling pathways regulated by specific chemicals [30]. We chose protocols based on chemical differentiation because of its recapitulation of natural development. The obtained MSN cultures were analyzed for their efficiency in developing ER stress (Figure 1).

## 2. Results

### 2.1. Generation and Characterization of iPSC Lines of a Patient with HD

As a result of reprogramming blood mononuclear cells in accordance with the published protocol [31], we obtained 34 iPSC clones from a patient with Huntington’s disease in the symptomatic stage.

**Figure 2:**
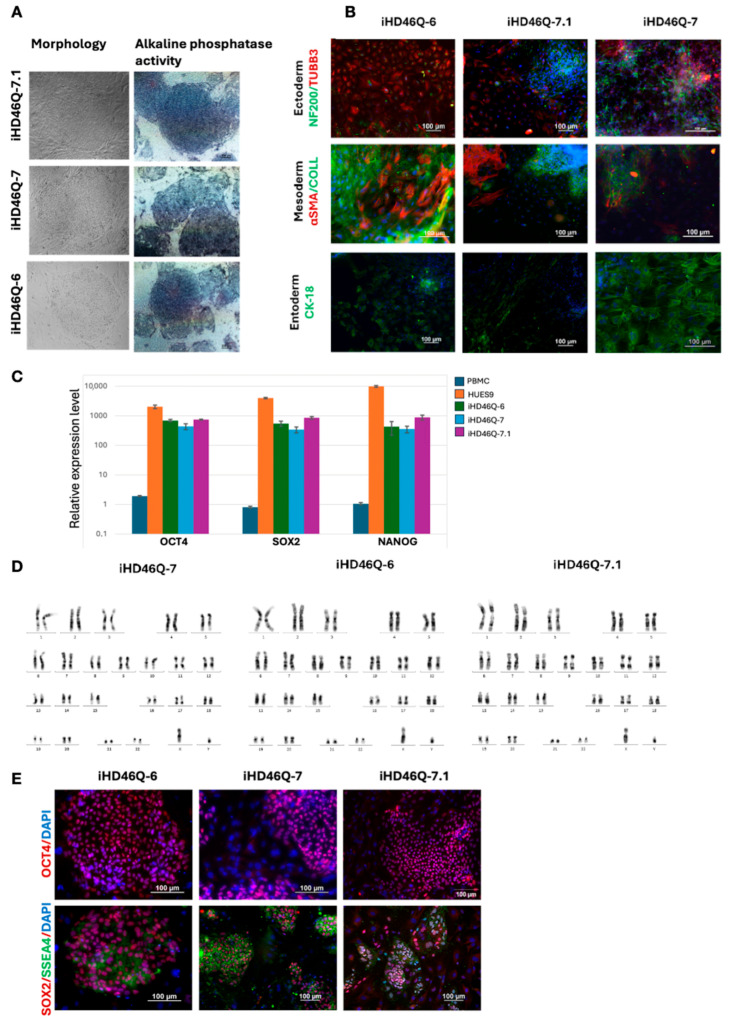
Characterization of iHD46Q iPSC cell lines. (**A**) Morphology of iPSC colony and alkaline phosphatase detection. (**B**) Immunofluorescence staining of iHD46Q-6, iHD46Q-7 and iHD46Q-7.1 cell cultures after in vitro spontaneous differentiation: αSMA and Collagen (mesoderm markers); TUBB3 and NF200 (ectoderm markers); CK-18 (endoderm marker). (**C**) Quantitative RT-PCR analysis of expression of pluripotency markers (NANOG, OCT4, SOX2). (**D**) Karyotype analysis (46,XY). (**E**) Immunofluorescence staining for pluripotency markers OCT4, SOX2, SSEA-4. All scale bars—100 μm.

After characterization of the iPSC lines, we chose three of them for further experimentation: iHD46Q-6, iHD46Q-7 and iHD46Q.7.1. Immunofluorescent staining of markers of pluripotency and spontaneous differentiation, as well as quantitative RT-PCR, proved the pluripotent status of these cell lines (Figure 2).

**Figure 3:**
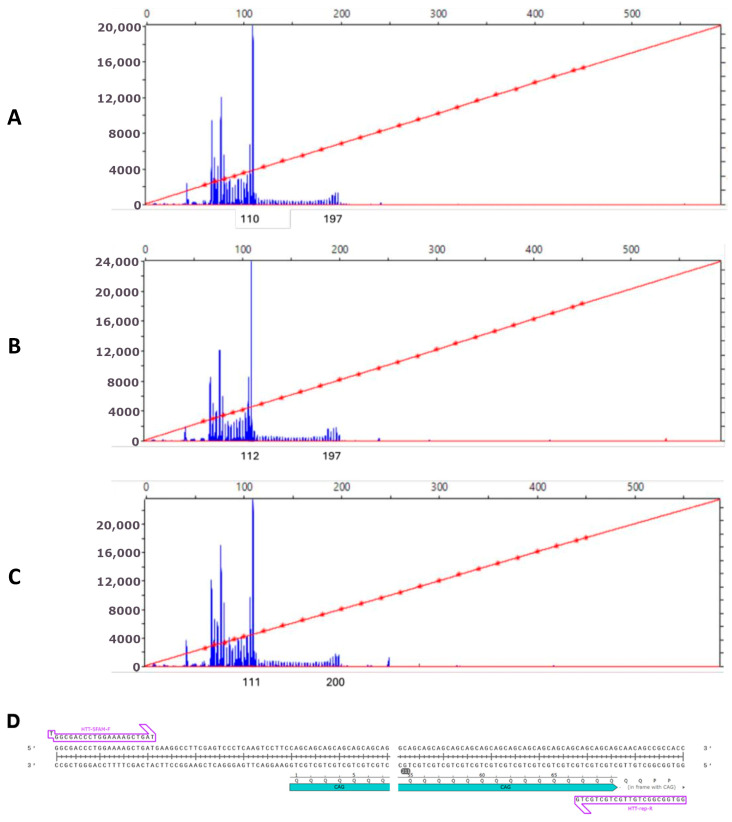
Analysis of CAG repeats length in iPSC lines by capillary electrophoresis: (**A**) iHD46Q-6 (48 CAG); (**B**) iHD46Q-7 (48 CAG); (**C**) iHD46Q-7.1 (49 CAG); (**D**) location of primers on the target sequence (arrows). The blue region marks the CAG-repeat sequence in the first exon of the *HTT* gene.

To determine the CAG repeats length in *HTT* gene, we conducted fragment analysis (Figure 3). Normally, the number of CAG repeats is 6-26, but expansion of the CAG repeats for 36 or more causes the pathological phenotype manifestation. The length of CAG in mutated *HTT* allele in the obtained iHD46Q iPSC lines turned out to be 48 repeats, and the second normal allele contained 15 CAG.

### 2.2. Generation of Transgenic iPSC Lines Expressing XBP1-TagRFP Biosensor

**Figure 4:**
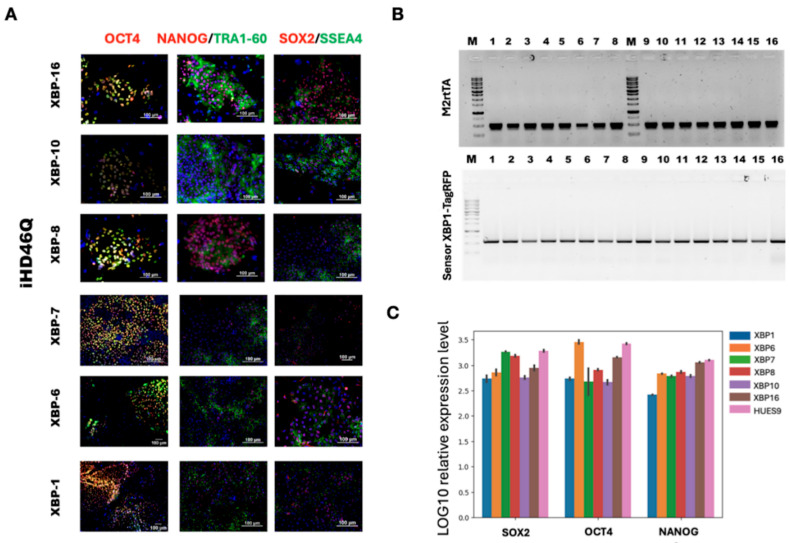
Characterization of the iHD46Q-7.1-based transgenic iPSC lines with XBP1-TagRFP construction in *AAVS1* locus, and tetracycline transactivator M2rtTA, integrated by Sleeping Beauty method. (**A**) Morphology of iPSC colonies and alkaline phosphatase staining. (**B**) PCR-analysis of XBP1-TagRFP and M2rtTA transgene integration. (**C**) qPCR-analysis of expression of pluripotency markers *SOX2*, *OCT4* and *NANOG*.

**Figure 5:**
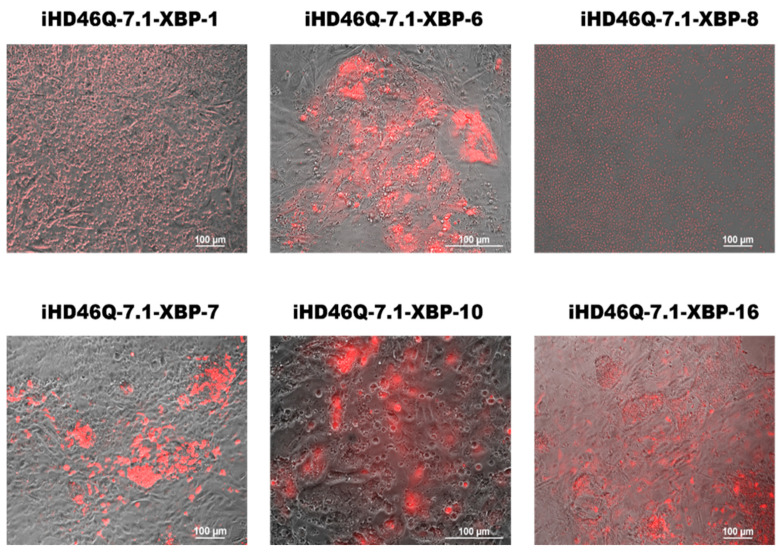
XBP1-TagRFP sensors produce red signals in response to the ER stress inducer tunicamycin, in transgenic iPSC clones XBP-1, XBP-6, XBP-8, XBP-7, XBP-10 and XBP-16 of iHD46Q-7.1 cell line.

Genetically encoded biosensors are a powerful tool for studying cell processes in living cells. We used the XBP1-TagRFP biosensor to observe ER stress in patient-specific iPSC-derived neural cells. We integrated two types of transgenes into the genome using CRISPR/Cas9 and Sleeping Beauty methods in the iHD46Q-7.1 iPSC clone (Figures S1 and S2). Donor molecules for transgene integration into the *AAVS1* locus were generated as described earlier [22]. The transgene encoding M2rtTA transactivator was integrated using the Sleeping Beauty transposon vector. Both donor vectors contained antibiotic resistance genes, Geneticin Sulfate (G418, neomycin analog) resistance in XBP1-TagRFP donor, and puromycin resistance in M2rtTA donor, for further cell selection. The working concentrations of selective antibiotics (200 ng/mL for puromycin and 30 μg/mL for Geneticin Sulfate (G418)) were determined during titration on the iHD46Q-7.1. We selected the concentration at which cells without antibiotic resistance genes died within 5 days. Neomycin and puromycin-resistant iPSC clones were mechanically selected and transferred onto the feeder layer for further cultivation. To confirm the presence of integrated genetic constructs, PCR analysis with specific primers was performed (Figure 4). The transgenes encoding XBP1-TagRFP sensor and M2rtTA transactivator were found in all selected iPSC clones. The transgenic iPSCs demonstrate red fluorescence when culturing in the presence of tunicamycin, an ER stress inducer (Figure 5).

Thus, obtained transgenic iPSC allows the visualization of activation of one of the ER stress pathways involving XBP1 splicing by IRE1 under the induction of ER stress activators, such as tunicamycin. We further tested whether the ER stress sensor was activated in neuronal derivatives of these transgenic iPSCs, i.e., whether stress developed along the XBP1-IRE1 pathway in neurons.

### 2.3. Testing Capability of Obtained iPSC Lines to Differentiate into Neural Derivatives

We differentiated transgenic iPSC lines into MSNs: XBP-1, XBP-6, XBP-7, XBP-8 and XBP-16 lines as well as the control K6-4f iPSC line of a healthy donor [32]. Differentiation was performed using three differentiation protocols: protocols No. 1 by Grigor’eva et al. (2020), No. 2 by Fjodorova et al. (2018), and No. 3 by Stanslowsky et al. (2016) [25,26,27].

**Figure 6:**
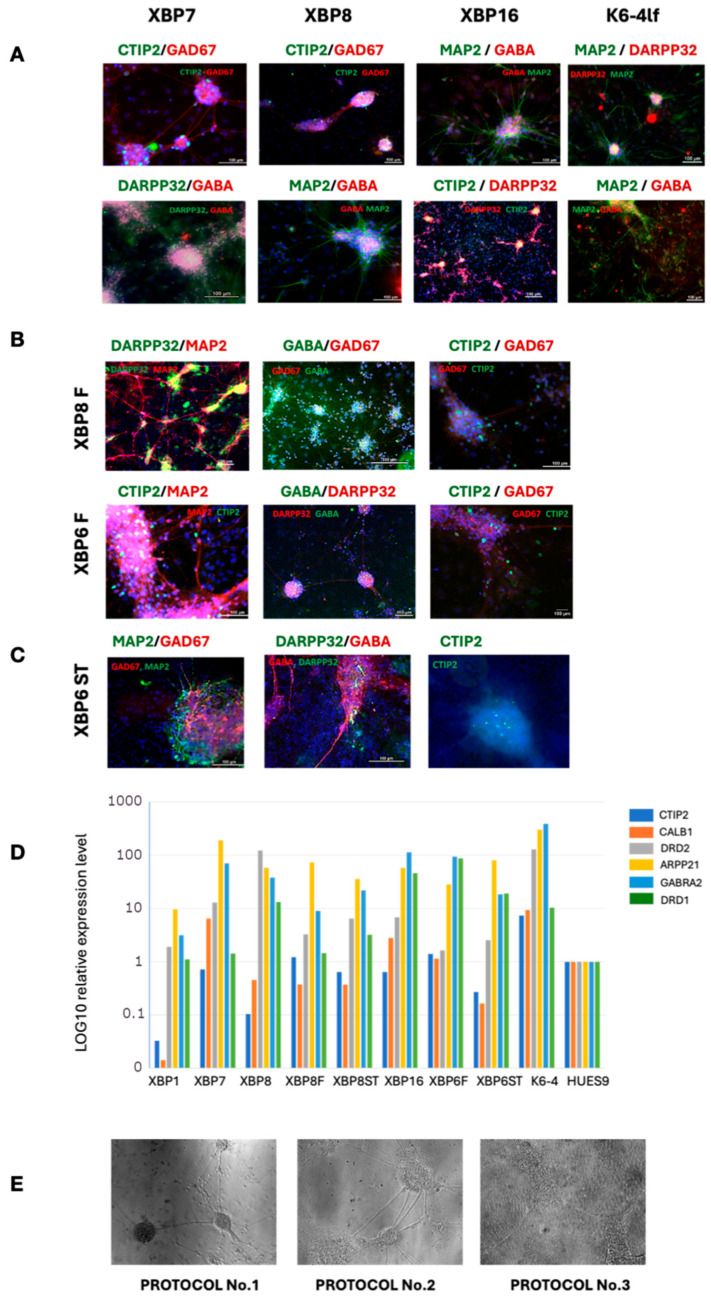
Transgenic iPSC-derived neuronal cultures expressing markers of medium spiny neurons. Immunofluorescent staining with specific antibodies to MSN markers: (**A**) obtained by the differentiation protocol No. 1, (**B**) obtained by the differentiation protocol No. 2, (**C**) obtained by the differentiation protocol No. 3. (**D**) Quantitative RT-PCR analysis of MSN markers expression level in neuronal cultures. (**E**) Morphology of obtained neuronal cultures following protocols No. 1–3.

The obtained neuronal cultures are listed in Table 1. Derived neural cultures were further analyzed using immunofluorescent staining with specific antibodies to MSN markers GABA, CTIP2, DARPP32 and GAD67 (Figure 6) and the common neural markers TUBB3 and MAP2 [33].

Protocol No. 1 yielded neuronal cultures XBP-1, XBP-7, XBP-8 and XBP-16 of a patient with HD, and K6-4 of a healthy donor. According to the quantitative PCR testing, the XBP-7 and XBP-16 lines are characterized by high expression of striatum marker gene *ARPP21* and a marker of GABAergic neurons *GABRA2*. XBP-16 cells also have high expression of the second type of dopamine receptor gene *DRD2* (Figure 6D). The K6-4 line has a higher expression of all marker genes compared to the patient-specific lines; however, it does not express *CTIP2*. Immunofluorescence staining of neuronal markers also revealed the presence of MAP2, GABA and GAD67, as well as the specific marker DARPP32. At the same time, there are also a small number of CTIP2-expressing neurons in K6-4 culture—their proportion is very low compared to the total number of cells. This is also reflected in qPCR analysis (Figure 6D).

The XBP-6 and XBP-8 lines successfully underwent differentiation into the MSNs according to the first protocol and were selected as control populations to compare efficiency of two other differentiation protocols.

According to the second protocol [26], we obtained the XBP-6F and XBP-8F neural cultures after 40 days of differentiation. XBP-8F cells express the *ARPP21* gene, whereas the XBP-6F cells demonstrate a high expression level of other important markers, such as *GABRA2* and *DRD1,* characterizing the presence of MSNs with type 1 dopamine receptors in the cell population. Immunofluorescence staining also confirmed the presence of GABAergic neurons expressing DARPP32 and CTIP2.

According to the third protocol [25], we obtained two cultures of neurons, XBP-6ST and XBP-8ST, after 40 days of differentiation. Both cultures express markers of GABAergic striatum neurons (Figure 6D), differing only in the type of dopamine receptors (DRD1, DRD2). In comparison with the culture of neurons obtained using the other two protocols, XBP-6ST and XBP-8ST cultures had a smaller number of neuron-like cells, with a larger proportion of cells demonstrating a high proliferative potential. Immunofluorescence staining of the neuronal cultures showed the presence of GABAergic neuronal markers (GABA, GAD67) and a low representation of specific MSN markers (DARPP32, CTIP2) (Figure 6C).

In general, the protocol No. 1 proved to be more effective in terms of time—mature GABAergic neurons with expression of MSN markers DARPP32, ARPP21, CALB1, DRD1 were obtained on day 33–35 of differentiation [27], although only a small proportion of CTIP2-expressing neurons were present in the culture. In addition, protocol No. 1 allows long-term cultivation of MSN precursors and controlled onset of terminal differentiation (by a sparse plating of the precursors, 1:10), which is convenient for planning large-scale experiments. However, not all the neuronal cultures obtained according to Protocol No. 1 turned out to be successful. For example, XBP-1 cell culture demonstrated a low level expression of both GABAergic and MSN marker genes. Protocol No. 2 allowed us to obtain more cells with the neural phenotype expressing markers of GABAergic striatal neurons, in contrast to protocol No. 3.

In general, all obtained neuronal cultures demonstrated a higher level of MSN marker expression compared to pluripotent cells, but this was not always sufficient enough to be considered relevant. The morphology of the obtained cell cultures suggests the presence of other cell types, such as glial cells, which may influence the results of future experiments.

### 2.4. Detection of XBP1-Mediated ER Stress in MSNs

ER stress is one of the hallmarks that were observed within HD. mHTT interferes with the ubiquitin–proteasome system that leads to inhibition of protein degradation, including ERAD. Thus, accumulation of unfolded proteins in ER causes ER stress and UPR [14]. In mammalian cells, there are three essential signaling pathways for ER stress activation mediated by IRE1, PERK, and ATF6 [20].

UPR activation was observed in animal models of HD, and the products of UPR-associated genes, such as ATF6, BIP, and CHOP, were up-regulated. Moreover, it was shown that striatal neurons are very sensitive to ER stress as their PERK pathway is deficient [34].

The role of the IRE1 pathway is not clear, but it is known that striatum-targeted inhibition of XBP1 in mice improves HD phenotype [18]. In addition, ectopic expression of IRE1 leads to its self-activation and aggregation of mHTT [35]. Treatment of neuronal cells with an ER stress inducer, tunicamycin, increased mHTT aggregation through IRE1 activation. Recent studies showed that kinase activity of IRE1 was necessary to stimulate mHTT aggregation [36]. So, it is supposed that IRE1-mediated cascade activity could be a potential marker of ER stress in HD. Here, we analyzed the endoribonuclease activity of IRE1, causing XBP1 splicing, in iPSC-derived neurons using XBP1-TagRFP biosensor for ER stress. The biosensor was activated when a spliced form of XBP1 (sXBP1) appeared.

**Figure 7:**
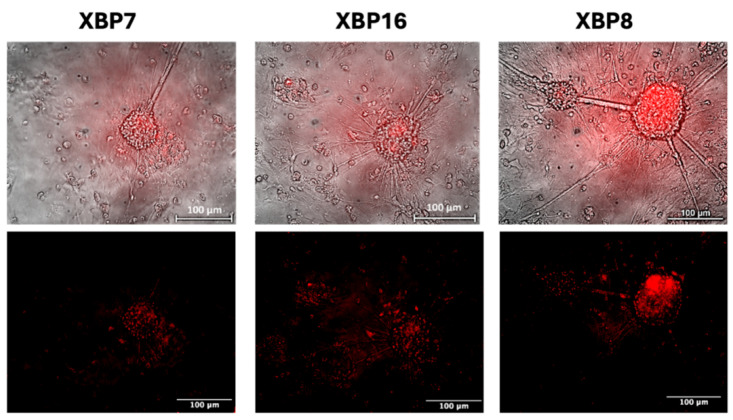
ER stress sensor activation in neural cultures after adding tunicamycin. Red signals demonstrate the activity of the XBP1-TagRFP biosensor.

We cultured neurons for two days in an antioxidant-free medium to induce self-protecting systems of neurons, as a complete neuronal medium contains antioxidants such as ascorbic acid, vitamin E, vitamin E acetate, superoxide dismutase, catalase, and glutathione. We observed no fluorescent signals of the biosensor under normal cultivation conditions of neural cells as well as in the antioxidant-free medium. However, the addition of the ER stress inducer, tunicamycin, to the medium leads to red fluorescence (Figure 7).

**Figure 8:**
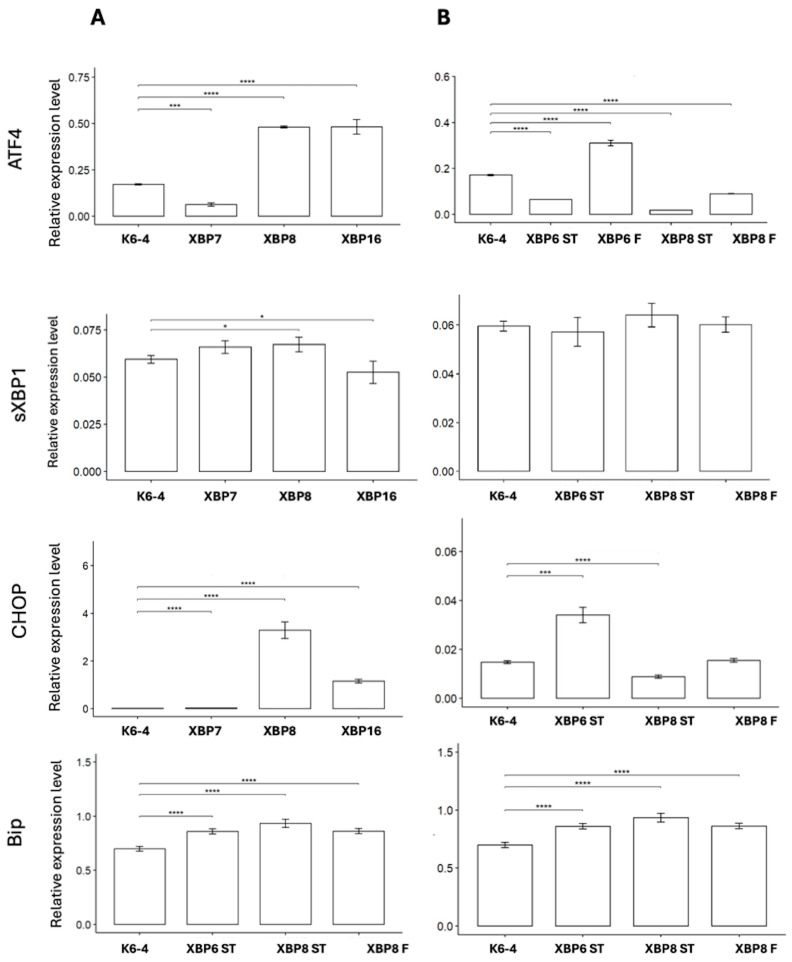
Changes in the expression level of ER stress marker genes. (**A**) Expression level of *ATF4*, *sXBP1*, *CHOP*, *BIP* in neuronal cultures derived by protocol No. 1. (**B**) Expression level of *ATF4*, *sXBP1*, *CHOP*, *BIP* in neuronal cultures derived by protocol No. 2–3. *—*p* < 0.05; ***—*p* < 0.01; ****—*p* < 0.001.

The absence of sensor activity led us to assess the level of expression of *sXBP1* and other markers of ER stress, such as *ATF4*, *CHOP* and *BIP*. We analyzed the expression of these genes in neuronal cultures, differentiated according to protocols No. 1–3. Our results show activity of *ATF4* and *CHOP* in HD neuronal cultures compared to K6-4 (Figure 8). The results indicate that ER stress in neuronal cultures derived by protocols No. 2 and 3 is actually activated in an IRE1-independent manner.

We consolidated data on the *ATF4*, *sXBP1*, *CHOP* and *BIP* gene expression levels compared to the healthy control K6-4 in Table 2. A general tendency towards ER stress development was observed in neuronal cultures obtained using the protocol No. 1. Some cases of up-regulation of *ATF4*, *sXBP1* and *CHOP* were also observed in XBP-6F, XBP-6ST and XBP-8F. The expression of *BIP* was present in all neuronal cultures.

It is worth noting that there are several deviations among the results from neuronal cultures obtained using different protocols: most analyzed cultures based on the protocol No. 1 showed up-regulation of the studied genes. The other neuronal cultures have opposite results; for example, XBP-8F, XBP-6ST and XBP-8ST demonstrated down-regulation of *ATF4*.

## 3. Discussion

Nowadays, iPSC-based models of various diseases, including neurodegenerative diseases, are commonly widespread. Among the known mechanisms of HD, ER stress and the UPR have been recognized and investigated for a long time. It has been pointed out that mHTT is able to interfere with ER stress activation, as shown in animal models of HD and in postmortem tissue samples [37,38]. To analyze the reproduction of a phenotypic characteristic such as ER stress development in a relevant cell type, we differentiated iPSC lines from an HD patient and a healthy donor to MSNs. The iPSC lines and MSNs express biosensors for ER stress related to the IRE1 pathway. Thus, derived cell models can be used to study the pathological processes of HD in terms of the UPR and ER stress, and to test the effects of potential drugs.

Actually, numerous treatment strategies and methods aimed at ER stress have been developed. According to neurodegenerative diseases, it was shown that overexpression of XBP1 may be neuroprotective in the animal models of Parkinson’s disease [39], while its silencing leads to a decreasing in neuron loss in mice models of HD [18]. Chemical chaperones proved to be effective to reduce ER stress development in mice models of HD [40]. Nevertheless, existing potential therapeutic means are under consideration: it is necessary to assess their effects and mechanisms based on the novel findings. So, repurposing currently available chemicals to stabilize aberrant UPR signaling could be a possible way to treat HD [2].

In the course of our work, we attempted to determine whether the IRE1 pathway plays a crucial role in MSN disturbances at the early stages of HD.

The existing protocols of MSN differentiation have various limitations. To overcome these challenges and obtain average and common results, we used three protocols [25,26,27]. It turned out that the resulting cultures of neural derivatives had no significant differences—in particular, the expression of specific markers of MSNs was observed in all of them, with the proportion of mature MSNs varying from culture to culture within each protocol. But some markers demonstrated low expression levels across all neuronal cultures (*CTIP*, *CALB1*). Immunofluorescent staining proved the most relevant neuronal cultures of iPSC-derived MSNs—XBP-7, XBP-16, XBP-8, XBP-8F. Summarizing the results of morphology and expression analysis of MSN markers, it can be concluded that protocols No. 1 and No. 2 were the most suitable for obtaining cultures with an acceptable proportion of MSNs.

All used protocols are based on dual SMAD inhibition, but they influence different components of the signaling pathways. As it is known, early inhibition of the WNT/β-catenin pathway is required for the telencephalic induction of the neural plate. Therefore, protocols No. 1–2 include purmorphamine, whereas protocol No. 3 uses IWP2 to mediate neural differentiation through embryoid structures. Further specific differentiation of the lateral ganglionic eminence (LGE) is carried out by the TGFβ signaling pathway. However, protocols No. 1 and 2 mediate this process through the implementation of Activin A, which induces a regional patterning towards the LGE, whereas the third protocol applies TGFβ3. Activin A is added immediately after dual SMAD inhibition according to the protocol No. 2, and further terminal differentiation and maturation of MSNs is regulated by adding BDNF and GDNF. The first protocol allows cultivation of neuronal precursors for some time before inducing their terminal differentiation by adding BDNF, and Activin A is added only during terminal differentiation [27]. However, it is worth noting that an accurate time point of terminal differentiation triggering is necessary. According to the third protocol, terminal MSN differentiation is regulated by adding TGFβ inductors as well as factors of neuronal maturation (BDNF, GDNF).

All in all, protocols No. 1–2 combined two approaches to LGE patterning: via the SHH pathway (using SHH agonist Purmorphamine) and via the Activin signaling pathway. The third protocol involved a different approach—embryoid body formation, triggering neuroectodermal differentiation by regulating SHH and WNT pathways, and applying TGFβ3 instead of Activin A. Consequently, neuronal cultures obtained according to the third protocol were the most unstable during differentiation—there were several differentiation runs before we were able to derive morphologically acceptable neuronal cultures. The first and second protocols provided neuronal cultures with required neural-like morphology, with all derived neuronal cells expressing specific MSN markers that varied insignificantly.

Regardless, for analyzing the development of ER stress, we used neuronal cultures obtained using every considered protocol.

Several approaches were used to evaluate the effect of the mutation in the *HTT* gene on ER stress development in the culture of medium spiny neurons. First of all, we tested the development of ER stress along the XBP1-IRE1 pathway using a genetically encoded biosensor. Endoribonuclease IRE1 is activated during ER stress and produces the spliced mRNA form of the XBP1 mRNA. IRE1 activation results in the XBP1-TagRFP biosensor red fluorescence in neuronal cultures [20,21]. In our study, we failed to observe any red signal in neuronal cultures, either in complete medium or in the antioxidant-free condition. To check whether ER stress develops in the iPSC-derived neuronal cultures, we analyzed the expression of ER stress marker genes by quantitative RT-PCR. To measure the expression levels of *sXBP1* (spliced), *CHOP* and *ATF4*, we used six lines of neural cultures obtained from the patient’s iPSCs: XBP-7, XBP-8, XBP-16 (obtained according to protocol No. 1), XBP-8F (obtained according to protocol No. 2), XBP-6ST, XBP-8ST (obtained according to protocol No. 3). Most neuronal cultures derived by protocol No. 1 demonstrated increased expression levels of *ATF4*, *CHOP*, *BIP* and even *sXBP1*. This may indicate that ER stress takes place in the obtained neuronal cultures, though other pathways (PERK or ATF6) may be involved in the process [41]. On the other hand, the presence of the *sXBP1* in some neuronal cultures along with the absence of red fluorescence may indicate that the activity of IRE1 is not enough to be detected by the XBP1-TagRFP biosensor. In addition, since neuronal cultures are heterogeneous, they may contain both neuronal and glial cells. This may be beneficial for modeling complex interactions within cell populations, which prevent the development of stress-related conditions in the MSNs.

## 4. Materials and Methods

### 4.1. Ethical Statement

The study was approved by the Ethics Committee of the Center for New Medical Technologies, Novosibirsk, Russia (protocol No. 21, 2017). The HD patient signed informed consent.

### 4.2. Generation of iPSC Lines by Reprogramming PBMCs of an HD Patient

Mononuclear cells were isolated from 16 mL of blood by phase separation using Histopaque-1077 (Merck KGaA, Darmstadt, Germany). Reprogramming to a pluripotent state was performed according to a previously published protocol using reprogramming episomal vectors (Addgene IDs #41855-58, #41813-14) [31]. Primary colonies were picked manually and transferred onto a feeder layer in the iPSC cultivation medium (DMEM/F12, KoSR 15%, 2 mM GlutaMAX-I, 0.1 mM NEAA, 100 U/mL penicillin–streptomycin (all Thermo Fisher Scientific, Waltham, MA, USA), 0.1 mM 2-mce (Sigma-Aldrich, Darmstadt, Germany), and 10 ng/mL bFGF (SCI Store, Moscow, Russia)). Characterization of the pluripotent cell clones was performed as described earlier [32]. The primers and antibodies used for the analysis of the pluripotency marker expression are listed in Table 3 and Table 4. The obtained iPSC lines were registered in the hPSCreg database with the following accession numbers: ICGi059-A for iHD46Q7.1, ICGi059-B for iHD46Q6 and ICGi059-C for iHD46Q7 (https://hpscreg.eu/cell-line/ICGi059-A, accessed on 31 July 2025).

### 4.3. Assessment of CAG Repeat Tract Length in HTT Gene

Genome DNA was amplified using BioMaster HS-Taq PCR-Color Kit (2×) (Biolabmix, Novosibirsk, Russia), 0.5 pmol/μL FAM-labeled Forward primer and 0.5 pmol/μL Reverse primer (Table 5) and 10% DMSO on Thermocycler T100 (Bio-Rad Laboratories, Singapore). Program parameters: 95 °C 5 min; 35 cycles—95 °C 15 s, 63 °C 15 s, 72 °C 25 s; 72 °C 25 min. Capillary electrophoresis was performed in the ICG Joint Centre for Genome Studies using capillary genetic analyzer Nanophore 05 (Syntol, Moscow, Russia), size standard CD-450. Data were analyzed using SequenceScanner software v2.0 (Thermo Fisher Scientific, Waltham, MA, USA).

To calculate the exact number of CAG repeats, we subtract 61 nucleotides from the length of the PCR product, including the size of the primers and the regions surrounding the repeats, and divide the result by 3.

### 4.4. Generation of Transgenic iPSC Lines with XBP1-TagRFP Biosensor

Transgenic iPSC clones expressing the XBP1-TagRFP ER stress sensor and the M2rtTA transactivator (reverse tetracycline-controlled transactivator) were generated as described earlier [21]. The XBP1-TagRFP ER stress sensor with the puromycin resistance gene were integrated into the AAVS1 locus using CRISP/Cas9 technology and a donor construct—pXBP1-TagRFP-ERSS-donor (Figure S1) [22]. M2rtTA transactivator and the neomycin resistance gene were integrated using the SB100X Sleeping Beauty system and M2rtTA-donor construct (Figure S2). In addition, the plasmid encoding CRISPR/Cas9 and sgRNA (Table 3), based on the px458 plasmid (Addgene ID #48138), was used to cut the genome in the *AAVS1* locus, and the transposase-encoding plasmid (Addgene ID #65487) was used for the Sleeping Beauty system. The transfection procedure was performed on the Neon Transfection System (Thermo Fisher Scientific, Waltham, MA, USA) with the program 1100 V, 30 ms, 1 pulse. After electroporation, cells were plated onto a layer of mitotically inactivated MEFs in antibiotic-free iPSC culture medium containing 2 µM thiazovivin (Sigma-Aldrich, Darmstadt, Germany). A total of 48 h after transfection, the cells were selected using 30 µg/mL geneticin (G418) sulfate (Santa Cruz Biotechnology, Dallas, TX, USA) for 72 h. A day after G418 withdrawal, 200 ng/mL puromycin (Santa Cruz Biotechnology, Dallas, TX, USA) was added to the culture medium for 3–4 days. The surviving double-resistant colonies were mechanically transferred to separate 48-well plates and analyzed for transgene integration by PCR with primers (Table 3), as previously described [22].

Pluripotency of the transgenic clones was verified by the RT-qPCR analysis of expression of pluripotency markers OCT4, NANOG and SOX2 (Table 3), immunofluorescence staining for pluripotency markers OCT4, SSEA-4, TRA1-60 and NANOG (Table 4) and spontaneous differentiation and immunofluorescence analysis of markers of three germ layers (Table 4). Karyotyping of the new clones was performed using DAPI banding as described in [21].

### 4.5. Spontaneous Differentiation

iPSC colonies were detached using 0.15% Type IV Collagenase (Thermo Fisher Scientific, Waltham, MA, USA) and seeded on 10 cm dishes coated with 1% agarose in DMEM/F-12 medium containing 10% FBS, 0.1 mM NEAA, 2 mM GlutaMax, 100 U/mL penicillin–streptomycin (all Thermo Fisher Scientific, Waltham, MA, USA) and cultivated for two weeks. Embryoid bodies were plated on Matrigel-treated 8-well Chambered Coverglass plates (Thermo Fisher Scientific, Waltham, MA, USA) and cultivated for 7 days. After this period the immunofluorescence staining was performed (Table 4).

### 4.6. Immunofluorescence Staining

Cells grown in wells were prefixed by adding 100 µL of 4% PFA (Sigma-Aldrich, Darmstadt, Germany) per well. After removing the medium with the prefixer, 200 µL of 4% PFA was added to the well and left for 10 min. Cells were washed twice with PBS for 15 min and permeabilized in 0.5% Triton X-100 (Sigma-Aldrich, Darmstadt, Germany) for 30 min. After washing, the fixed cells were incubated in PBS with 1% BSA (Sigma-Aldrich, Darmstadt, Germany) for 30 min. Next, primary antibodies were diluted in 1% BSA (Table 4), added to the wells and left overnight at 4 °C. The unbound antibodies were washed twice for 15 min with PBS, and secondary antibodies were added (Table 4) in 1% BSA for 1.5 h. For visualization, the nuclei were stained with a DAPI solution (1 μg/mL). All procedures were performed at room temperature. Visualization was performed using a Nikon Eclipse Ti-E fluorescence microscope (Nikon, Tokyo, Japan) and NIS Elements Advanced Research v. 4.30 software.

### 4.7. Directed Differentiation iPSC into Medium Spiny Neurons

**Figure 9:**
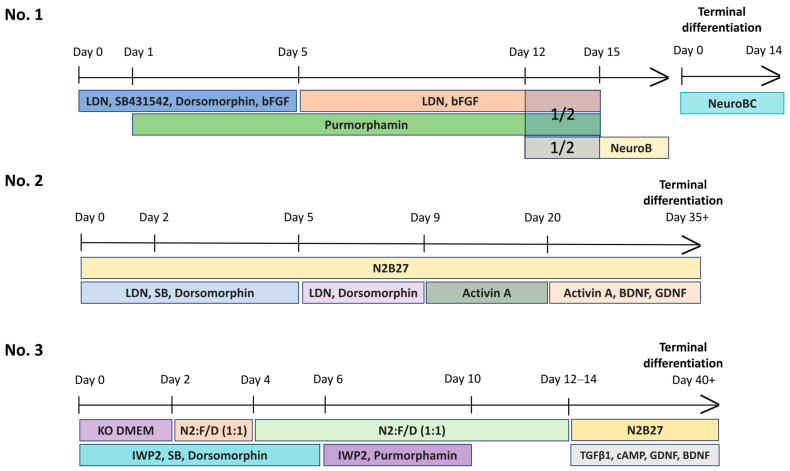
Schematic representation of the steps of the protocols for directed differentiation of iPSCs into medium spiny neurons.

The schematic presentation of the MSN differentiation protocols used in the study is shown in Figure 9.

#### 4.7.1. Protocol No. 1 [27]

The protocol by Grigor’eva et al. was used to derive cultures of MSN XBP-1, XBP-7, XBP-8 and XBP-16.

iPSCs were cultured for 2 passages in Essential 8 medium (Thermo Fisher Scientific, Waltham, MA, USA) on Matrigel-GFR extracellular matrix (Corning, New York, NY, USA). Once reaching confluency 80–90% (Day 0), the medium was changed to neuronal differentiation medium (NDM): F12/DMEM:Neurobasal (Thermo Fisher Scientific, Waltham, MA, USA) 2:1, 1x N2 Supplement (Thermo Fisher Scientific, Waltham, MA, USA), 100 ng/mL LDN193189 hydrochloride (Sigma-Aldrich, Darmstadt, Germany), 8 µM SB431542 (StemRD, Burlingame, CA, USA), 2 µM dorsomorphin (Sigma-Aldrich, Darmstadt, Germany) and 4 ng/mL bFGF, 1× pen/strep. From day 1 to day 13, 0.6 µM purmorphamine (Stemgent, Beltsville, MD, USA) was added. On day 5, SB431542 and dorsomorphin were removed from the medium. On day 13, the cells were disaggregated by Accutase Cell Dissociation Reagent (Thermo Fisher Scientific, Waltham, MA, USA) and segregated 1:2, adding 2 µM Thiazovivin (Sigma-Aldrich, Darmstadt, Germany) to the medium. The next day, the medium was changed to a 1:1 mix of NDM (-SB,-Dorsomorphin) and NeuroB (Neurobasal, 1× B-27 Supplement (Thermo Fisher Scientific, Waltham, MA, USA), 20 ng/mL recombinant human BDNF (PeproTech, Cranbury, NJ, USA), 1.1 mM ascorbic acid (Sigma-Aldrich, Darmstadt, Germany) and 1 × pen/strep. On day 15, the medium was completely changed to NeuroB and the precursors of neurons were cultured in monolayer, passaged every 5–7 days. For terminal differentiation to MSN, the precursors were disaggregated using Accutase and seeded at a density 2 × 10^4^ cells/cm^2^ onto Matrigel-coated plates and cultivated in terminal differentiation medium for 10–15 days (Neurobasal, 19 B-27, 19 pen/strep, 20 ng/mL BDNF, 1.1 mM ascorbic acid, 25 ng/mL recombinant human/murine/rat Activin A (PeproTech, Cranbury, NJ, USA) with 10 ng/mL recombinant human CNTF (Biolegend, San Diego, CA, USA) and 0.5 mM dbcAMP (PeproTech, Cranbury, NJ, USA).

#### 4.7.2. Protocol No. 2 [26]

Differentiation protocol of iPSCs to MSN according to Fjodorova et al. was used to derive MSN cultures XBP-6F, XBP-8F.

iPSCs were cultured on Matrigel-GFR extracellular matrix (Corning, New York, NY, USA) in the Essential 8 medium (Thermo Fisher Scientific, Waltham, MA, USA) for 2–3 passages (Figure 9). After they reached a density of 80–90%, the medium was changed to N2B27 (day 0): 50% DMEM/F-12: 50% Neurobasal, 1× B-27 Supplement (Thermo Fisher Scientific, Waltham, MA, USA), GlutaMAX-1 (Thermo Fisher Scientific, Waltham, MA, USA), 1% penicillin/streptomycin, 0.1% 50 mM β-ME, 10 µM SB431542 (StemRD, Burlingame, CA, USA), 100 nM LDN-193189 hydrochloride (Sigma-Aldrich, Darmstadt, Germany), 200 nM Dorsomorphin. From day 5 N2B27 was supplemented only with 100 nM LDN193189 and 200 nM Dorsomorphin. From day 9, the N2B27 medium was supplemented only with activin A (Biolegend, San Diego, CA, USA), and the cells were passaged every 5–7 days in a 1:10 ratio, using 0.02% 0.5 M EDTA. The terminal differentiation was conducted from day 21 by adding to N2B27 medium activin A, 10 ng/mL BDNF, 10 ng/mL GDNF. The medium was replaced every four days to 35 ± 3 days.

#### 4.7.3. Protocol No. 3 [25]

Differentiation of iPSCs was performed according to the Stanslowsky et al.’s protocol to derive MSN cultures XBP-6ST, XBP-8ST.

iPSCs were cultured on Matrigel-GFR extracellular matrix (Corning, New York, NY, USA) in the Essential 8 medium (Thermo Fisher Scientific, Waltham, MA, USA) for 2–3 passages (Figure 9). After reaching the density 70–90%, colonies were separated using 0.5 mM EDTA and transferred to Petri dishes (⌀ 20 cm) coated with agarose, and cultured in knockout DMEM medium with the addition of 20% knockout serum substitute, 0.1 mM β-mercaptoethanol, 1% 100 × penicillin/streptomycin, 1% 200 mm of L-glutamine and 0.1 mM of interchangeable amino acids, 5 microns of Y-27632 (StemRD, Burlingame, CA, USA), 1 µM Dorsomorphin (Sigma-Aldrich, Darmstadt, Germany), 10 µM SB-431542 (StemRD, Burlingame, CA, USA) and 1 µM IWP2 (Sigma–Aldrich, Darmstadt, Germany) for the formation of embryoid bodies. Two days later, the medium was replaced with a 1:1 mixture of KSR and N2 media (DMEM/F-12 with 1:100 N2 (Thermo Fisher Scientific, Waltham, MA, USA) and 1% penicillin/streptomycin/L-glutamine) with ROCK, DM, SB and IWP2. On day 4, the medium was replaced with medium N2 using the same combination of small molecules. On days 6 and 8, the N2 medium was changed with the addition of 0.6 µg/mL of Purmorphamine (Stemgent, Beltsville, MD, USA) and 1 µg/mL of IWP2. On days 10 and 12, N2 medium was added without any small molecules. On day 14, the embryoid bodies were transferred to wells coated with Matrigel-GFR extracellular matrix (Corning, New York, NY, USA) and cultured in N2B27 maturation medium (DMEM/F-12 containing 1:200 N2, 1:100 B27 without vitamin A (Thermo Fisher Scientific, Waltham, MA, USA), 20 ng/mL of BDNF, 10 ng/mL of GDNF and 1 ng/mL of TGF-β3, 0.5 mM of dibutyryl cyclic AMP (PeproTech, Cranbury, NJ, USA). The medium was changed every four days.

### 4.8. Analysis of MSN and ER Stress Marker Genes Expression by RT-qPCR

RNA was isolated using Trizol (Thermo Fisher Scientific). Reverse transcription of 1 µg of RNA was performed using M-MuLV-RH reverse transcriptase (Biolabmix, Russia). qRT-PCR reactions were run on a QuantStudio 5 Real-Time PCR System (Applied Biosystems) with BioMaster HS-qPCR SYBR Blue 2× (Biolabmix). CT values were normalized to B2M. The expression of MSN markers was assessed using the ΔΔCT method, with human embryonic stem cell lines HUES9 serving as a reference [42]. ER stress marker genes were analyzed using the ΔCT method. The K6-4 MSN culture obtained in the study was used as a control for ER stress markers estimation. Primers are presented in Table 3.

## 5. Conclusions

In the study, we created a patient-specific cell model of HD, which enriched the collection of models of the disease and allowed us to expand our knowledge of the pathogenesis mechanisms and to search for potential therapeutic agents. Furthermore, we developed an improved model for the targeted study of the IRE1 signaling pathway in ER stress. Experiments showed that IRE1-mediated ER stress development may not occur in iPSC-derived MSN cultures, despite the presence of other markers of ER stress.

## Figures and Tables

**Table 1:** Derived neural cultures and source iPSC lines.

Protocol	Transgenic iPSC Line	MSN Culture Name
No. 1	iHD46Q-7.1-XBP-1	XBP-1
No. 2	iHD46Q-7.1-XBP-6	XBP-6F
No. 3		XBP-6ST
No. 1	iHD46Q-7.1-XBP-7	XBP-7
No. 1	iHD46Q-7.1-XBP-8	XBP-8
No. 2		XBP-8F
No. 3		XBP-8ST
No. 1	iHD46Q-7.1-XBP-16	XBP-16
No. 1	K6-4f (healthy donor)	K6-4

**Table 2:** Expression pattern of ER stress marker genes in iPSC-derived neuronal cultures.

Neuronal Culture	*ATF4*	*sXBP1*	*CHOP*	*BIP*
XBP-7	up		up	up
XBP-8	up	up	up	up
XBP-16	up	up	up	up
XBP-6F	up			up
XBP-8F	down	up	up	up
XBP-6ST	down	up	up	up
XBP-8ST	down			up

**Table 3:** Oligonucleotides and primers for PCR and RT-PCR detection of pluripotency, MSN, ER stress markers and transgene integrations.

Gene		Sequence
Markers of ER stress	*ATF4*	TTCTCCAGCGACAAGGCTAAGG/CTCCAACATCCAATCTGTCCCG
	*CHOP*	GGTATGAGGACCTGCAAGAGGT/CTTGTGACCTCTGCTGGTTCTG
	*Bip (GRP78)*	CTGTCCAGGCTGGTGTGCTCT/CTTGGTAGGCACCACTGTGTTC
	*sXBP1*	TCTGCTGAGTCCGCAGCAG/GAAAAGGGAGGCTGGTAAGGAAC
Markers of pluripotency	*NANOG*	CAGCCCCGATTCTTCCACCAGTCCC/CGGAAGATTCCCAGTCGGGTTCACC
	*SOX2*	GCTTAGCCTCGTCGATGAAC/AACCCCAAGATGCACAACTC
	*OCT4*	CTTCTGCTTCAGGAGCTTGG/GAAGGAGAAGCTGGAGCAAA
Markers of MSN	*GABRA2*	CCCAATGCACTTGGAGGATTTCC/AGAGCCATCAGGAGCAACCTGT
	*ARPP21*	CCTACCTCAACCACGCAACAGT/CCTGTTGACCAGACAAGACTGG
	*CTIP2 (BCL11B)*	CTCCCTTTGGATGCCAGTGTCA/GGCTCCAGGTAGATGCGGAAG
	*DRD1*	TGGTCTGTGCTGCCGTTATCAG/CAATCTCAGCCACTGCCTTCCA
	*DRD2*	CAATACGCGCTACAGCTCCAAG/GGCAATGATGCACTCGTTCTGG
	*CALB1*	TTTCCTGCTGCTCTTCCGATGC/GCTCCTCAGTTTCTATGAAGCCA
Detection of transgene insertions	*XBP1-TagRFP*	CCGGACCACTTTGAGCTCTAC/AGGCGCACCGTGGGCTTGTAC
	*M2rtTA*	CAGCCGGAACACGGCGGCATC/ACACCGTGCGTTTTATTCTGTC
Reference	*B2M*	TAGCTGTGCTCGCGCTACT/ TCTCTGCTGGATGACGTGAG
sgRNA for *AAVS1*	*AAVS1*	CACCGGTCACCAATCCTGTCCCTAG/ AAACCTAGGGACAGGATTGGTGACC

**Table 4:** Antibodies used in the study.

Marker	Antibody	Dilution	Company
Markers of ectoderm	Rabbit IgG anti-NF200	1:1000	Sigma-Aldrich Cat. # N4142
	Mouse IgG2a anti-Tubulin β 3 (TUBB3)/Clone: TUJ1	1:1000	BioLegend Cat. # 801201
Markers of mesoderm	Mouse IgG2a anti-αSMA	1:100	Dako Cat. # M0851
	Mouse IgG1 anti-Collagen IV	1:100	LifeSpan Biosciences Cat # LS-C79603
Markers of endoderm	Mouse IgG1 anti-CK18	1:200	Abcam, ab668
Markers of pluripotency	Mouse IgGγ3 anti-SSEA4	1:200	Abcam, ab16287
	Rabbit IgG anti-SOX2	1:500	Cell Signaling, 3579
	Mouse IgGγ2b anti-OCT4	1:50	Santa Cruz, sc-5279
	Mouse IgGγ1 anti-NANOG	1:50	Santa Cruz, sc-293121
	Rabbit IgG anti-NANOG	1:500	REPROCELL, RCAB004P-F
Secondary antibodies	Goat anti-Mouse IgG (H + L) Secondary Antibody, Alexa Fluor 568	1:400	Thermo Fisher Scientific, A11031
	Goat anti-Rabbit IgG (H + L) Highly Cross-Adsorbed Secondary Antibody, Alexa Fluor 488	1:400	Thermo Fisher Scientific, A11008
	Goat anti-Mouse IgG2b CrossAdsorbed Secondary Antibody, Alexa Fluor 568	1:400	Thermo Fisher Scientific, A-21141
	Goat anti-Mouse IgG1 Cross-Adsorbed Secondary Antibody, Alexa Fluor™ 488	1:400	Thermo Fisher Scientific, A-21121
	Goat anti-Mouse IgG3 CrossAdsorbed Secondary Antibody, Alexa Fluor 488	1:400	Thermo Fisher Scientific, A21151

**Table 5:** Primers used for fragment analysis.

Name of Primer	Sequence
Forward (FAM-labeled)	[FAM-AH]TGGCGACCCTGGAAAAGCTGAT
Reverse	GGTGGCGGCTGTTGCTGCTGCTG

## Data Availability

All data is presented within the article. The characterization of the newly obtained iPSC lines is also available in the Human Pluripotent Stem Cell Registry (https://hpscreg.eu) with the following accession numbers: ICGi059-A for iHD46Q7.1, ICGi059-B for iHD46Q6 and ICGi059-C for iHD46Q7 (https://hpscreg.eu/cell-line/ICGi059-A, accessed on 31 July 2025).

## Supplementary Materials

The following supporting information can be downloaded at https://www.mdpi.com/article/10.3390/ijms26188930/s1.

## Abbreviations

The following abbreviations are used in this manuscript:

iPSC	Induced pluripotent stem cell
HD	Huntington’s disease
ER	Endoplasmic reticulum
ERAD	Endoplasmic reticulum-associated protein degradation system
UPR	Unfolded protein response

## References

[1] Saudou F., Humbert S. (2016). The Biology of Huntingtin. Neuron.

[2] Makeeva V.S., Dyrkheeva N.S., Lavrik O.I., Zakian S.M., Malakhova A.A. (2023). Mutant-Huntingtin Molecular Pathways Elucidate New Targets for Drug Repurposing. Int. J. Mol. Sci..

[3] Caron N.S., Dorsey E.R., Hayden M.R. (2018). Therapeutic Approaches to Huntington Disease: From the Bench to the Clinic. Nat. Rev. Drug Discov..

[4] Ferguson M.W., Kennedy C.J., Palpagama T.H., Waldvogel H.J., Faull R.L.M., Kwakowsky A. (2022). Current and Possible Future Therapeutic Options for Huntington’s Disease. J. Cent. Nerv. Syst. Dis..

[5] Kaye J., Reisine T., Finkbeiner S. (2022). Huntington’s Disease IPSC Models—Using Human Patient Cells to Understand the Pathology Caused by Expanded CAG Repeats. Fac. Rev..

[6] Liu C., Oikonomopoulos A., Sayed N., Wu J.C. (2018). Modeling Human Diseases with Induced Pluripotent Stem Cells: From 2D to 3D and Beyond. Development.

[7] Kaye J.A., Finkbeiner S. (2013). Modeling Huntington’s Disease with Induced Pluripotent Stem Cells. Mol. Cell. Neurosci..

[8] Galimberti M., Nucera M.R., Bocchi V.D., Conforti P., Vezzoli E., Cereda M., Maffezzini C., Iennaco R., Scolz A., Falqui A. (2024). Huntington’s Disease Cellular Phenotypes Are Rescued Non-Cell Autonomously by Healthy Cells in Mosaic Telencephalic Organoids. Nat. Commun..

[9] Marei H.E. (2025). Stem Cell and Synthetic Embryo Models: Advances, Applications, and Ethical Considerations. Stem Cell Rev. Rep..

[10] Liu W., Kennington L.A., Rosas H.D., Hersch S., Cha J.H., Zamore P.D., Aronin N. (2008). Linking SNPs to CAG Repeat Length in Huntington’s Disease Patients. Nat. Methods.

[11] Martin D.D.O., Kay C., Collins J.A., Nguyen Y.T., Slama R.A., Hayden M.R. (2018). A Human Huntingtin SNP Alters Post-Translational Modification and Pathogenic Proteolysis of the Protein Causing Huntington Disease. Sci. Rep..

[12] Claassen D.O., Corey-Bloom J., Dorsey E.R., Edmondson M., Kostyk S.K., Ledoux M.S., Reilmann R., Rosas H.D., Walker F., Wheelock V. (2020). Genotyping Single Nucleotide Polymorphisms for Allele-Selective Therapy in Huntington Disease. Neurol. Genet..

[13] Handsaker R.E., Kashin S., Reed N.M., Tan S., Lee W.S., McDonald T.M., Morris K., Kamitaki N., Mullally C.D., Morakabati N.R. (2025). Long Somatic DNA-Repeat Expansion Drives Neurodegeneration in Huntington’s Disease. Cell.

[14] Duennwald M.L., Lindquist S. (2008). Impaired ERAD and ER Stress Are Early and Specific Events in Polyglutamine Toxicity. Genes. Dev..

[15] Leitman J., Ulrich Hartl F., Lederkremer G.Z. (2013). Soluble Forms of PolyQ-Expanded Huntingtin Rather than Large Aggregates Cause Endoplasmic Reticulum Stress. Nat. Commun..

[16] Shenkman M., Eiger H., Lederkremer G.Z. (2015). Genesis of ER Stress in Huntington’s Disease. Endoplasmic Reticulum Stress Dis..

[17] Chen Y., Brandizzi F. (2013). IRE1: ER Stress Sensor and Cell Fate Executor. Trends Cell Biol..

[18] Vidal R.L., Figueroa A., Court F.A., Thielen P., Molina C., Wirth C., Caballero B., Kiffin R., Segura-Aguilar J., Cuervo A.M. (2012). Targeting the UPR Transcription Factor XBP1 Protects against Huntington’s Disease through the Regulation of FoxO1 and Autophagy. Hum. Mol. Genet..

[19] Le Q.G., Kimata Y. (2021). Multiple Ways for Stress Sensing and Regulation of the Endoplasmic Reticulum-Stress Sensors. Cell Struct. Funct..

[20] Iwawaki T., Akai R., Kohno K., Miura M. (2004). A Transgenic Mouse Model for Monitoring Endoplasmic Reticulum Stress. Nat. Med..

[21] Yarkova E.S., Grigor’eva E.V., Medvedev S.P., Tarasevich D.A., Pavlova S.V., Valetdinova K.R., Minina J.M., Zakian S.M., Malakhova A.A. (2024). Detection of ER Stress in IPSC-Derived Neurons Carrying the p.N370S Mutation in the GBA1 Gene. Biomedicines.

[22] Ustyantseva E.I., Medvedev S.P., Vetchinova A.S., Minina J.M., Illarioshkin S.N., Zakian S.M. (2019). A Platform for Studying Neurodegeneration Mechanisms Using Genetically Encoded Biosensors. Biochemistry.

[23] Arber C., Precious S.V., Cambray S., Risner-Janiczek J.R., Kelly C., Noakes Z., Fjodorova M., Heuer A., Ungless M.A., Rodríguez T.A. (2015). Activin A Directs Striatal Projection Neuron Differentiation of Human Pluripotent Stem Cells. Development.

[24] Telezhkin V., Schnell C., Yarova P., Yung S., Cope E., Hughes A., Thompson B.A., Sanders P., Geater C., Hancock J.M. (2016). Forced Cell Cycle Exit and Modulation of GABAA, CREB, and GSK3β Signaling Promote Functional Maturation of Induced Pluripotent Stem Cell-Derived Neurons. Am. J. Physiol. Cell Physiol..

[25] Stanslowsky N., Reinhardt P., Glass H., Kalmbach N., Naujock M., Hensel N., Lübben V., Pal A., Venneri A., Lupo F. (2016). Neuronal Dysfunction in IPSC-Derived Medium Spiny Neurons from Chorea-Acanthocytosis Patients Is Reversed by Src Kinase Inhibition and F-Actin Stabilization. J. Neurosci..

[26] Fjodorova M., Li M. (2018). Robust Induction of DARPP32-Expressing GABAergic Striatal Neurons from Human Pluripotent Stem Cells. Methods Mol. Biol..

[27] Grigor’eva E.V., Malankhanova T.B., Surumbayeva A., Pavlova S.V., Minina J.M., Kizilova E.A., Suldina L.A., Morozova K.N., Kiseleva E., Sorokoumov E.D. (2020). Generation of GABAergic Striatal Neurons by a Novel IPSC Differentiation Protocol Enabling Scalability and Cryopreservation of Progenitor Cells. Cytotechnology.

[28] Le Cann K., Foerster A., Rösseler C., Erickson A., Hautvast P., Giesselmann S., Pensold D., Kurth I., Rothermel M., Mattis V.B. (2021). The Difficulty to Model Huntington’s Disease in Vitro Using Striatal Medium Spiny Neurons Differentiated from Human Induced Pluripotent Stem Cells. Sci. Rep..

[29] Golas M.M. (2018). Human Cellular Models of Medium Spiny Neuron Development and Huntington Disease. Life Sci..

[30] Telias M. (2023). Neural Differentiation Protocols: How to Choose the Correct Approach. Neural Regen. Res..

[31] Okita K., Yamakawa T., Matsumura Y., Sato Y., Amano N., Watanabe A., Goshima N., Yamanaka S. (2013). An Efficient Nonviral Method to Generate Integration-Free Human-Induced Pluripotent Stem Cells from Cord Blood and Peripheral Blood Cells. Stem Cells.

[32] Malakhova A.A., Grigor’eva E.V., Pavlova S.V., Malankhanova T.B., Valetdinova K.R., Vyatkin Y.V., Khabarova E.A., Rzaev J.A., Zakian S.M., Medvedev S.P. (2020). Generation of Induced Pluripotent Stem Cell Lines ICGi021-A and ICGi022-A from Peripheral Blood Mononuclear Cells of Two Healthy Individuals from Siberian Population. Stem Cell Res..

[33] Straccia M., Barriga G.G.D., Sanders P., Bombau G., Carrere J., Mairal P.B., Vinh N.N., Yung S., Kelly C.M., Svendsen C.N. (2015). Quantitative High-Throughput Gene Expression Profiling of Human Striatal Development to Screen Stem Cell-Derived Medium Spiny Neurons. Mol. Ther. Methods Clin. Dev..

[34] Maity S., Komal P., Kumar V., Saxena A., Tungekar A., Chandrasekar V. (2022). Impact of ER Stress and ER-Mitochondrial Crosstalk in Huntington’s Disease. Int. J. Mol. Sci..

[35] Lee H., Noh J.Y., Oh Y., Kim Y., Chang J.W., Chung C.W., Lee S.T., Kim M., Ryu H., Jung Y.K. (2012). IRE1 Plays an Essential Role in ER Stress-Mediated Aggregation of Mutant Huntingtin via the Inhibition of Autophagy Flux. Hum. Mol. Genet..

[36] Bae D., Jones R.E., Piscopo K.M., Tyagi M., Shepherd J.D., Hollien J. (2022). Regulation of Blos1 by IRE1 Prevents the Accumulation of Huntingtin Protein Aggregates. Mol. Biol. Cell.

[37] Brandstaetter H., Kruppa A.J., Buss F. (2014). Huntingtin Is Required for ER-to-Golgi Transport and for Secretory Vesicle Fusion at the Plasma Membrane. DMM Dis. Models Mech..

[38] Jin H., Komita M., Aoe T. (2018). Decreased Protein Quality Control Promotes the Cognitive Dysfunction Associated with Aging and Environmental Insults. Front. Neurosci..

[39] Valdés P., Mercado G., Vidal R.L., Molina C., Parsons G., Court F.A., Martinez A., Galleguillos D., Armentano D., Schneider B.L. (2014). Control of dopaminergic neuron survival by the unfolded protein response transcription factor XBP1. Proc. Natl. Acad. Sci. USA.

[40] Keene C.D., Rodrigues C.M., Eich T., Chhabra M.S., Steer C.J., Low W.C. (2002). Tauroursodeoxycholic acid, a bile acid, is neuroprotective in a transgenic animal model of Huntington’s disease. Proc. Natl. Acad. Sci. USA.

[41] Ong G., Ragetli R., Mnich K., Doble B.W., Kammouni W., Logue S.E. (2024). IRE1 signaling increases PERK expression during chronic ER stress. Cell Death Dis..

[42] Cowan C.A., Klimanskaya I., McMahon J., Atienza J., Witmyer J., Zucker J.P., Wang S., Morton C.C., McMahon A.P., Powers D. (2004). Derivation of Embryonic Stem-Cell Lines from Human Blastocysts. N. Engl. J. Med..

