# Supplementary figures and images for: Generating a Cell Model to Study ER Stress in iPSC-Derived Medium Spiny Neurons from a Patient with Huntington’s Disease
Source: Int J Mol Sci. 2025 Sep 13;26(18):8930. doi: 10.3390/ijms26188930 (PMC12469972; doi:10.3390/ijms26188930)

Created with SnapGene®

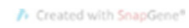

Created with SnapGene®

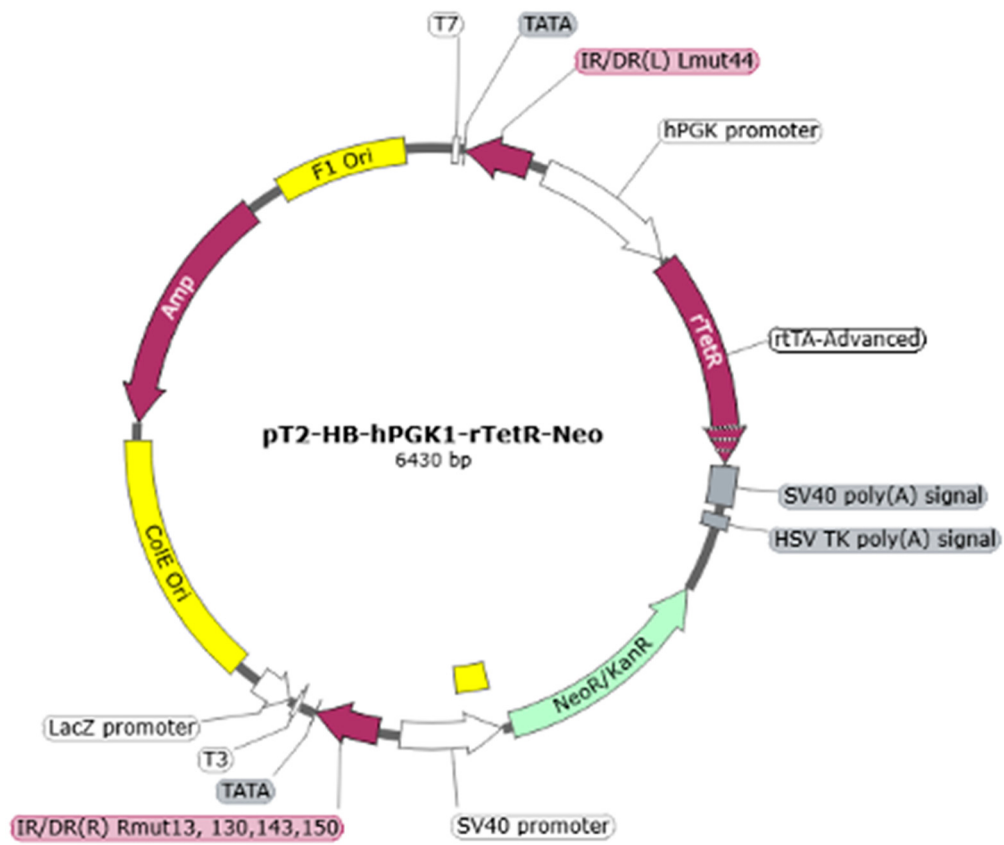

Figure S2. M2rtTA-donor construct map

Supplement: Supplementary file 1 [file ijms-26-08930-s001.zip › ijms-3828437-supplementary.pdf]
